# Nursing insights into CAR T-cell therapy for systemic lupus erythematosus: Highlights of a standardized nursing system for CAR T-cell therapy

**DOI:** 10.1515/rir-2026-0005

**Published:** 2026-03-30

**Authors:** Yujie Yang, Lingling Gao, Kunling Wang, Xiangfeng Li

**Affiliations:** Department of Rheumatology and Clinical Immunology, Department of Internal Medicine, Peking Union Medical College Hospital; Peking Union Medical College and Chinese Academy of Medical Science; Key Laboratory of Rheumatology and Clinical Immunology, Ministry of Education, Beijing, China

Dear Editor,

Chimeric antigen receptor (CAR) T-cell therapy holds promise for treating autoimmune diseases, such as systemic lupus erythematosus (SLE).^[1]^ Research has demonstrated the effectiveness of CAR T-cell therapy in patients with refractory SLE, prompting further evaluation of the effectiveness of CD19 CAR T cells in treatment‐resistant SLE patients.^[2]^

SLE patients are often diagnosed with multiple comorbidities related to the multiorgan involvement of SLE and treatment toxicity. Nursing plans involving CAR T-cell therapy for SLE patients are usually multidisciplinary and individually tailored.

CAR T-cell therapy is a complex process that involves several key steps, and careful monitoring of adverse events is crucial. However, challenges remain in nursing practice, including assessment, symptom management, patient education, care coordination, and psychosocial support. Consequently, this study aimed to analyze the effectiveness of nursing management processes for CAR T-cell therapy in SLE patients by utilizing the Health Failure Mode and Effects Analysis (HFMEA).

In this letter, we reported a study from Peking Union Medical College Hospital (PUMCH). This study comprised a small case series of six patients with treatment‐refractory SLE who were recruited from the Department of Rheumatology and Immunology of the PUMCH. Ethical approval was provided. All the patients were diagnosed with refractory SLE-associated thrombocytopenia, and all demonstrated prominent multiorgan involvement (lupus nephritis, 100%; musculoskeletal and cutaneous involvement, 66.7%; serositis, 33.3%). The median age of the participants was 27.5 years (IQR 21.8–34.8), and 66.7% were female.

**Figure 1:**
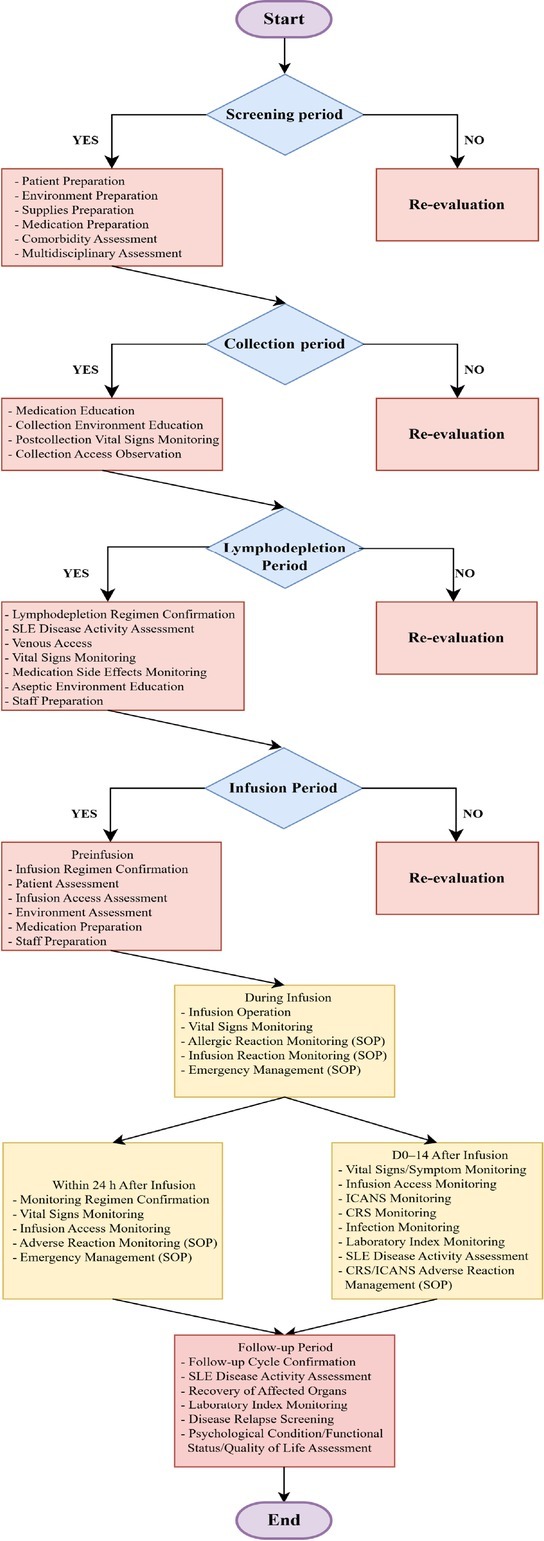
CAR T-cell therapy nursing management process for systemic lupus erythematosus (SLE) patients based on the HFMEA. HFMEA, Health Failure Mode and Effects Analysis.

We established an HFMEA team to evaluate the CAR T-cell therapy process for SLE patients. Through HFMEA, the team defined process failure modes, causes and consequences and formulated improvement measures and a nursing standard operating procedure (SOP) for CAR T-cell therapy in SLE patients. The key points of the SOP involved (1) infection prevention; (2) comorbidity assessment; (3) cell infusion; (4) management of infusion reactions; (5) toxicity monitoring and treatment; (6) emotional support; and (7) documentation and communication (Figure 1).

Unlike cancer patients, the SLE patients in this study were required to discontinue glucocorticoid and immunosuppressive therapy before CAR T-cell infusion. Notably, SLE patients often present with severe thrombocytopenia and are refractory, so withdrawn other therapeutic interventions leads to an extremely high risk of bleeding. Therefore, it is vital that nurses identify bleeding risks, provide health education on bleeding prevention, and implement key measures to prevent bleeding.

Cytokine release syndrome (CRS) is a common and potentially life-threatening toxicity of CAR T-cell therapy. Nurses play a crucial role in the early identification of CRS through close monitoring of vital signs and patient discomfort. Standardized assessment tools are used to evaluate the severity of CRS, which helps to make differential diagnosis and treatment decisions, including the administration of tocilizumab and glucocorticoids.^[3]^ One study patient developed agranulocytosis fever (38.2 °C; neutrophils, 200 per microlitre) on the 2nd day after infusion and was administered meropenem empirically. No haemodynamic changes were observed, and infection was ruled out; the patient was ultimately diagnosed with Grade 1 CRS, and after receiving supportive care, her fever resolved. When evaluating fever in CAR T-cell–treated SLE patients, it is essential to consider and thoroughly assess the following three key factors: the current activity status of SLE, the risk of infection associated with long-term immunosuppression, and the effects of CAR T-cell therapy.

Immune effector cell-associated neurotoxicity syndrome (ICANS) typically presents as neurotoxicity with symptoms such as difficulty reading, aphasia, and confusion. In severe cases, it can escalate to confusion, coma, seizures, movement disorders, and cerebral oedema. Nurses can perform regular neurological assessments to detect early signs of ICANS and monitor any changes in a patient’s mental status. Notably, none of the patients in this study developed ICANS.

Nurses can also offer emotional support throughout the treatment process. Nurses use the Hospital Anxiety and Depression Scale to assess patient’s psychological status and increase their confidence in the treatment, to eliminate pessimism, nervousness, and anxiety, and to encourage cooperation with the treatment with an optimistic mentality. Effective nurse communication and patient advocacy improve treatment outcomes and quality of life for individuals undergoing CAR T-cell therapy.^[4]^

The implementation of the nursing management process significantly reduced the safety risks associated with CAR T-cell therapy for the patients with SLE in this study. Only one patient experienced CRS, and there were no cases of severe CRS (grade 2 or higher). Furthermore, the incidence rate of ICANS was 0%. The patient’s outcome indicators tended to improve (Table 1). Moreover, the satisfaction rate was 100% at day 14 after infusion, reflecting high levels of satisfaction with nursing services.

**Table 1:** The effect of the CAR T-cell therapy nursing management process for SLE patients based on the HFMEA

Data	At admission	At day 14 after infusion
PhGA scores	1.8 (1, 2.4)	1.5 (0.6, 1.9)
SLEDAI scores	11.5 (8, 14.3)	6 (5.5, 10)
Laboratory studies		
C3 complement, g/L	0.6 (0.3, 0.8)	0.8 (0.6, 1)
C4 complement, g/L	0.1 (0, 0.2)	0.2 (0.2, 0.3)
SF-36 scores	43.3 (39.6, 59.7)	61.2 (60.3, 64)

PhGA, physician global assessment; SLEDAI, systemic lupus erythematosus disease activity index. SF-36, 36-item medical outcomes study short form survey; HFMEA, Health Failure Mode and Effects Analysis; CAR, Chimeric antigen receptor.

The HFMEA-based nursing management process, through proactive risk prevention and mitigation, standardized operational procedures, and full-cycle closed-loop management, significantly improved the clinical safety, nursing quality, and outcomes of CAR T-cell therapy for patients with SLE. This process provided a replicable and scalable empirical framework for nursing practice in cell therapy for special populations, offering significant clinical, educational, and practical value.
